# A novel application of quantile regression for identification of biomarkers exemplified by equine cartilage microarray data

**DOI:** 10.1186/1471-2105-9-300

**Published:** 2008-07-02

**Authors:** Liping Huang, Wenying Zhu, Christopher P Saunders, James N MacLeod, Mai Zhou, Arnold J Stromberg, Arne C Bathke

**Affiliations:** 1 Department of Statistics, 815 Patterson Office Tower, University of Kentucky, Lexington, Kentucky, 40508-0027, USA; 2 Department of Veterinary Science, Gluck Equine Research Center, Lexington, KY, 40546-0099, USA; 3 Document Forensics Laboratory, Department of Applied Information Technology, George Mason University, Fairfax, VA 22030, USA

## Abstract

**Background:**

Identification of biomarkers among thousands of genes arrayed for disease classification has been the subject of considerable research in recent years. These studies have focused on disease classification, comparing experimental groups of effected to normal patients. Related experiments can be done to identify tissue-restricted biomarkers, genes with a high level of expression in one tissue compared to other tissue types in the body.

**Results:**

In this study, cartilage was compared with ten other body tissues using a two color array experimental design. Thirty-seven probe sets were identified as cartilage biomarkers. Of these, 13 (35%) have existing annotation associated with cartilage including several well-established cartilage biomarkers. These genes comprise a useful database from which novel targets for cartilage biology research can be selected. We determined cartilage specific Z-scores based on the observed M to classify genes with Z-scores ≥ 1.96 in all ten cartilage/tissue comparisons as cartilage-specific genes.

**Conclusion:**

Quantile regression is a promising method for the analysis of two color array experiments that compare multiple samples in the absence of biological replicates, thereby limiting quantifiable error. We used a nonparametric approach to reveal the relationship between percentiles of M and A, where M is log_2_(R/G) and A is 0.5 log_2_(RG) with R representing the gene expression level in cartilage and G representing the gene expression level in one of the other 10 tissues. Then we performed linear quantile regression to identify genes with a cartilage-restricted pattern of expression.

## Background

DNA microarrays provide information about expression levels for thousands of genes simultaneously at the transcriptional level. It is being applied to determine how global (cell type, tissue, or organismal) differential transcription may affect biological systems. The development of microarray technology has motivated interest in their use for disease research and diagnosis. Many studies have attempted to find disease-specific biomarkers, a small subset of genes that distinguish normal tissue from diseased tissue. A wide variety of statistical methods have been applied to biomarker identification, including sparse logistic regression (SLogReg) [1], receiver operating characteristic (ROC) curve approach [2,3] and Gaussian process models [4]. However, most of these focus on disease classification, while far fewer studies have been done to identify tissue biomarkers or genes with a tissue-restricted pattern of expression. Genes with a high level of expression in one tissue compared to other tissue types in the body are likely to have corresponding tissue-restricted functional annotation. Further, loss of the functional product encoded by these genes will frequently be associated with tissue pathology. In general, the identification of tissue-specific biomarkers or genes with a tissue-restricted pattern of expression can provide important new insight into the biology of that tissue or the etiology/pathogenesis of diseases that impact that tissue.

Quantiles are measures of relative standing. For example, a student scoring at the *τ *th quantile on a standardized test means that he/she performs better than a proportion *τ *and worse than a proportion (1 - *τ*) of the reference group of students. For any 0 <*τ *< 1, *F*^-1^(*τ*) = inf{*x*: *F*(*x*) ≥ *τ*} is called the *τ *th quantile of the distribution *F*[5]. Quantile regression as introduced by Koenker and Bassett (1978) extends this idea to the estimation of conditional quantile functions modeling quantiles of the conditional distribution of the response variable as functions of observed covariates.

An ordinary least squares (OLS) regression models the relationship between covariates X and the conditional mean of the response variable Y given X = x. However, covariates X often influence the whole distribution of Y, not only the mean, thereby severely weakening OLS [6]. For example, a change in covariates may have an opposite effect on the high and low percentiles of Y. Unlike OLS, quantile regression methods offer a mechanism for estimating models across the full range of conditional quantile functions given X = x. Two models of quantile regression can be distinguished, depending on whether or not independent identically distributed (iid) error terms are assumed. We will call the model without assumption of iid error terms the non iid error model. In linear quantile regression, if the slopes of the regression lines are different for different quantiles, then the non iid error model is more appropriate [5]. Recently, Wang and He proposed a rank score test [7,8] for detecting differential gene expression by modeling and analyzing the quantiles of gene intensity distributions through probe-level measurements. Though also based on the quantile regression idea, Wang and He's method is otherwise not related to the approach presented here.

Fold change has been widely used in microarray experiments to identify genes with different expression levels between two types of samples (e.g., diseased versus normal tissue). A cut off of 2-fold up or down regulation has been chosen to define differential expression in most published studies [9,10]. However, the commonly used 2-fold change criterion does not take into account the magnitude of gene expression.

In this study, we propose an intensity-dependent linear quantile regression, using statistical and biological information to identify tissue-restricted patterns of gene expression. We demonstrate our methods on the analysis of cDNA microarray data to compare articular cartilage with ten different body tissues to identify genes with a cartilage-restricted pattern of expression representing potentially novel cartilage biomarkers. Chondrocytes are the only cell type in cartilage and they synthesize several proteins that are expressed in a highly tissue-restricted pattern, including type II procollagen and aggrecan core protein. Screening for novel genes that have a cartilage-restricted pattern of expression can expand our understanding of chondrocyte function and potentially improve our understanding of important diseases that involve cartilage, such as arthritis.

## Results and Discussion

### Implementation

**Figure 1:**
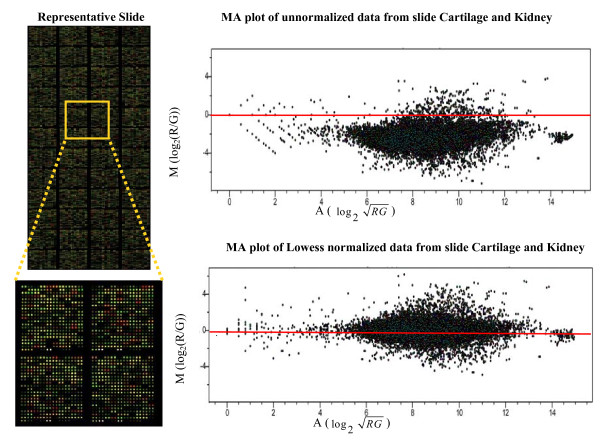
**MA plot to remove intensity dependent bias**. A MA plot was used to remove intensity dependent dye bias and array-specific effects, where M = log_2 _(R/G) and A = log_2_$\sqrt{RG}$. Above are two MA plots representative of the twenty cDNA microarray slides used for this study. The first plot illustrates unnormalized data and the second plot is the same data after Lowess (LOcally WEighted polynomial regreSSion) normalization.

**Figure 2:**
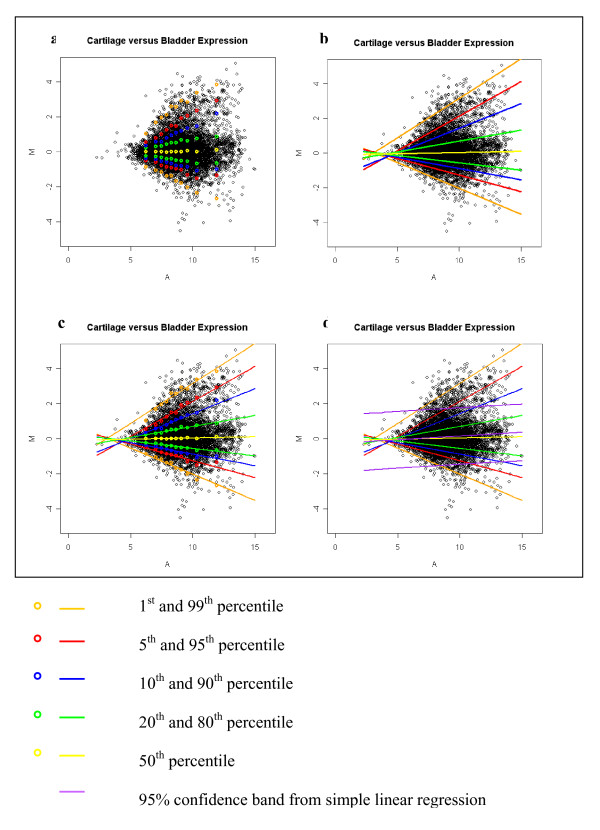
**Nonparametric approach to reveal the relationship between A and quantiles of M and linear quantile regression fitting**. a: A nonparametric approach to reveal the relationship between quantiles of M and A for the cartilage/bladder comparison; b: linear quantile regression using a linear term in model to fit the data for the cartilage/bladder comparison; c: goodness fit of the linear quantile regression; d: comparing the linear quantile regression with simple linear regression to show the inappropriateness of simple linear regression for this data set.

After scanning, the median intensities adjusted for background intensities of each pair of spots were Lowess (LOcally WEighted polynomial regreSSion) normalized for each individual slide. The MA plot shows that the intensity dependent bias had been removed after lowess normalization [11,12] (Figure 1). Because of some bad-flagged spots, the number of probesets available for analysis ranged from 9333 in the cartilage/lung comparison to 9411 in the cartilage/cerebellum comparison. For each comparison, a piecewise nonparametric approach was used to reveal the relationship between percentiles of M and A, where M = log_2 _(*R*/*G*) and A = log_2 _$\sqrt{RG}$ with R representing the gene expression level in cartilage and G representing the gene expression level in one of the other 10 tissues. The range of A was divided into 10 regions with a minimum of 900 probe sets and a maximum of 1000 probe sets in each region. The corresponding 1^st^, 5^th^, 10^th^, 20^th^, 50^th^, 80^th^, 90^th^, 95^th^, 99^th ^percentiles of M were calculated for each region of A. Scatter plots of the mean of A for each region and quantiles of M in the corresponding region were plotted. For the cartilage versus bladder comparison (Figure 2a), the scatter plot showed an approximate linear relationship between A and each of the considered conditional quantiles of M given A, with slight deviations from a linear relationship at the high intensities. Similar patterns were also observed in the other 9 tissue comparisons (data not shown). Since the scatter plots for different quantiles were not parallel, the non iid error quantile regression model is more reasonable. Hence for each comparison, linear quantile regression (containing intercept and a linear term) under the non iid error model [13,14] (Figure 2b) was fitted to the data. Generally, the fit was good, except for small deviations at extreme high intensities (Figure 2c). The corresponding nine conditional percentiles (1^st^, 5^th^, 10^th^, 20^th^, 50^th^, 80^th^, 90^th^, 95^th^, 99^th^) of M were estimated for each observed A. Observed M was compared to the estimated nine conditional percentiles of M, and a cartilage specific Z-score was calculated according to Table 1. The average Z score and standard deviation were also calculated. Genes were considered potential cartilage biomarkers if the observed values for M were above the estimated 95^th ^conditional percentile of M in all 10 of the cartilage/tissue comparisons analyzed (all Z-scores ≥ 1.96).

**Table 1:** Transforming quantiles of log_2_(R/G) to Z score.

Quantile of log2(R/G)	Z-score
observed log_2_(R/G) ≥ 99^th ^estimated quantile	2.57
99^th ^estimated quantile > observed log_2_(R/G) ≥ 95^th ^estimated quantile	1.96
95^th ^estimated quantile > observed log_2_(R/G) ≥ 90^th ^estimated quantile	1.44
90^th ^estimated quantile > observed log_2_(R/G) ≥ 80^th ^estimated quantile	1.04
80^th ^estimated quantile > observed log_2_(R/G) ≥ 50^th ^estimated quantile	0.39
50^th ^estimated quantile > observed log_2_(R/G) ≥ 20^th ^estimated quantile	-0.39
20^th ^estimated quantile > observed log_2_(R/G) ≥ 10^th ^estimated quantile	-1.04
10^th ^estimated quantile > observed log_2_(R/G) ≥ 5^th ^estimated quantile	-1.44
5^th ^estimated quantile > observed log_2_(R/G) ≥ 1^st^estimated quantile	-1.96
observed log_2_(R/G) < 1^st ^estimated quantile	-2.57

Transforming observed quantile of log_2_(R/G) into corresponding Z-score

### Cartilage biomarkers identified

**Figure 3:**
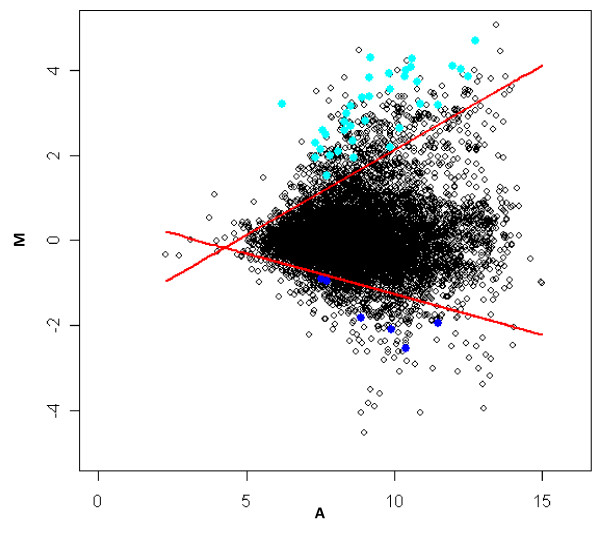
**Thirty seven probe sets identified with a high level of differential expression in cartilage and six probe sets with a low level of differential expression in cartilage in the cartilage/bladder comparison**. Two red straight lines correspond to estimated 95^th ^percentile and 5^th ^percentile of log_2_(R/G), respectively. In cartilage/bladder comparison, thirty seven probe sets (spots in cyan) fell above estimated 95^th ^percentile of log_2_(R/G) and six probe sets (spots in blue) fell below estimated 5^th ^percentile of log_2_(R/G).

Thirty-seven probe sets (cyan spots in Figure 3) were identified that exhibit expression above the 95^th ^conditional quantile in all 10 of the cartilage/tissue comparisons analyzed (Z-scores ≥ 1.96). Of these, 13 (35%) have existing annotation associated with cartilage including several well-established cartilage biomarkers (Table 2). BLAST hits for the remaining 24 probe sets (65%) in which the cartilage-specificity score was at least 1.96 in all 10 tissue comparisons have no reported sequence annotation associated with established functional roles in cartilage. From Table 2, we can also see that the means of the Z scores for these probe sets were high, with small standard deviations. In contrast, six probe sets (blue spots in Figure 3) exhibited expression levels below the 5^th ^conditional quantile in all 10 of the cartilage/tissue comparisons analyzed (Z-scores ≤ -1.96). These 6 probe sets represent the ones on this microarray (cDNAs from an equine articular cartilage library) with consistently low relative gene expression in cartilage compared to the other tissue types studied. With microarrays that contain probe sets for all genes in the genome of an organism, an analysis of the lowest quantiles should be useful in identifying genes with a near absence of expression in the reference tissue of interest.

**Table 2:** Cartilage-specific scores for genes with existing functional annotation linked to cartilage.

Gene Symbol	Gene Name	Cartilage-Specificity Score				
		
		Mean	S.D.	Low	High	Median
Hapln1	Hyaluronan and proteoglycan link protein 1	2.57	0	2.57	2.57	2.57
COMP	Cartilage oligomeric matrix protein	2.51	0.19	1.96	2.57	2.57
COL11A1	Collagen, type XI, alpha 1	2.51	0.19	1.96	2.57	2.57
AGC1	Aggrecan core protein	2.51	0.19	1.96	2.57	2.57
COL2A1	Collagen, type II, alpha 1	2.39	0.29	1.96	2.57	2.57
TNC	Tenascin C	2.33	0.32	1.96	2.57	2.57
PRG4	Proteoglycan 4	2.33	0.32	1.96	2.57	2.57
SOX9	SRY-box 9	2.20	0.32	1.96	2.57	1.96
ITGA10	Integrin, alpha 10	2.20	0.32	1.96	2.57	1.96

Cartilage-specificity scores for genes with existing functional annotation linked to cartilage and an established pattern of expression that is high in chondrocytes relative to many other cell types.

Probe sets with high fold change but very low intensities should be excluded. For example, a probe set might be reported with an intensity of 2 in bladder but 20 in cartilage, thus the fold change was 10. However, if the intensity reading in cartilage is low, then we cannot reliably identify this kind of probe set as one that is exhibiting cartilage-specific expression. For each chip, we calculate the 10^th ^percentile of averaged log intensities, denoted as A*. If a probe set's A value (averaged log intensity) was less than A*, we excluded it from the candidate list even when the M value (log fold change) for this probe set was very large. In other words, all 37 probe sets were selected from probe sets with values of A larger than A*. For one of the ten comparisons (cartilage vs. bladder), Figure 3 illustrates that A for all 37 probe sets was larger than 6 and M was larger than 1 which implies that the intensity reading in cartilage was at least greater than 64 after lowess normalization. Similar ranges of A values for these 37 probe sets were found in the other 9 cartilage/tissue comparisons. Taking COMP as an example in Table 3, we see that the intensity readings in cartilage were high and the relative expression differences between cartilage and each of the ten other tissues (fold change) were large. Similar ranges of intensity and relative expression differences were found with the other 36 probe sets. Therefore, data for these thirty-seven probe sets were interpreted as consistent with a cartilage-restricted pattern of expression.

**Table 3:** Intensities of COMP expression in all ten cartilage/tissue comparisons.

Tissue Comparison	Original Intensity		After Lowess Normalization		
		
	Cartilage	Tissue	A	M	Fold Change
Cartilage/Bladder	4797.70	214.07	9.17	7.63	194.01
Cartilage/Cerebellum	7860.70	947.79	10.36	5.35	40.79
Cartilage/Kidney	3249.25	264.16	8.69	5.89	59.30
Cartilage/Liver	5685.64	420.13	9.58	5.91	60.13
Cartilage/Lung	4494.31	166.70	8.77	5.83	56.89
Cartilage/Lymph node	10382.70	706.12	10.43	7.25	152.22
Cartilage/Muscle	6191.20	621.54	9.68	5.66	50.56
Cartilage/Placental villus	4256.93	238.23	8.83	7.27	154.34
Cartilage/Spleen	11358.35	806.09	10.52	7.18	145.01
Cartilage/Testis	8075.92	1774.16	11.04	4.60	24.25

Expression intensity data for cartilage oligomeric matrix protein (COMP) across the 10 tissue comparisons examined.

In this study, cartilage-specific scores were used in place of percentiles of M (Table 1). We have compared cartilage with ten other body tissues and have identified 37 probe sets with expression all above the 95^th ^percentile of M. However, with a larger number of tissue comparisons, the criterion of above the 95^th ^percentile of M in all tissue comparisons may be too stringent to identify a cartilage-restricted expression pattern. The idea of transforming percentiles of M into Z-scores and then choosing probe sets with a high average Z-score and low standard deviation makes the criterion more feasible to identify probe sets of interest. One of the advantages of the standardized Z-scores is that it is relatively simple to make adjustments that take the number of comparisons into account. The appropriate cutoff for average Z-score and standard deviation deserves further investigation.

Due to the fact that genes were classified as cartilage-specific only when they showed high relative expression in *all *10 tissue comparisons, the probability of falsely identifying a chance outlier as a cartilage-specific gene is rather low. Loguinov et al. [15] distinguish five different circumstances represented by "outliers": a gene with higher individual variability than the majority of genes; an outlier by chance; a sporadic technical or biological outlier; a systematic technical outlier (due to, for example, heteroscedasticity); or a systematic biological outlier due to differential expression. Our result is based on limited biological replicates, so it is important to distinguish between differentially-expressed probe sets and the other four types of outliers. We define genes as potential cartilage biomarkers if the observed values for M were above the estimated 95^th ^conditional percentile of M in *all *10 of the cartilage/tissue comparisons analyzed (allZ-scores ≥ 1.96). The probability that anyone of the thirty-seven probe sets identified would be due to the other four types of outliers in *all *10 of cartilage/tissue comparisons is very small. For example, if we assume that the probability of probe set being one of the other four types of outliers is 20% in one cartilage/tissue comparison, then the probability of this probe set being such an outlier in *all *10 cartilage/tissue comparisons is 0.2^10^, which is 1.024e-07, a rather small value.

### ▪ Feasibility and appropriateness of linear quantile regression

Volcano plots, which consider both statistical tests of differences between sample types (P value) and biological effects (fold change) are commonly used in microarray experiments to identify genes with different expression levels between two experimental groups. With microarray experiments in which the design requires comparisons between many experimental groups, the number of biological replicates can be constrained by logistical variables. For example, with the sample set analyzed in this study, the articular cartilage and eight of the comparative tissues were collected from a single donor while placental villous and testis samples were each collected from other donors. The absence of biological replicates made statistical inference (e.g., t-test) of expression differences between cartilage and the other 10 tissues impossible. In addition, a 2-fold change criterion does not take into account the varied magnitude of gene expression. Hence, quantile regression was used to determine quantiles of M conditional on A. Microarray data consists of thousands of probe sets. Dividing the range of A into several regions still makes each region have enough probe sets (corresponding to spots in the graph) to calculate the quantiles of M. Thus, the piecewise nonparametric method is feasible and appropriate to reveal the relationship between A and percentiles of M.

In this study, scatter plots showed percentiles above the 50^th ^percentile of M (99^th^, 95^th^, 90^th^, 80^th^) linearly increasing with A while percentiles below 50^th ^percentiles of M (1^st^, 5^th^, 10^th^, 20^th^) were linearly decreasing with A. Hence, linear quantile regression with a linear term was fitted to the data. Expression levels above the 95^th ^percentile were defined as cartilage-restricted expression. Thirty-seven probe sets were identified as exhibiting a cartilage-restricted pattern of expression. Within this group are widely recognized cartilage biomarkers, including genes encoding type II procollagen and aggrecan core protein. The presence of genes encoding these established cartilage biomarkers validate the linear quantile regression approach. However, we recognize that the expression pattern for the remaining genes that currently lack established functional annotation linked to cartilage needs to be confirmed with additional studies.

Simple linear regression (mean regression) should not be applied to these data since different quantiles of M behave differently (Figure 2d) and the iid error assumption (implying equal variances) which is used in simple linear regression is obviously violated. In Figure 2d, at medium and high intensities, the 95^th ^linear quantile regression line (red) was above the 95% confidence interval upper bound of the simple linear regression line (purple). As a result, the approach of fitting a linear regression and then calculating a 95% confidence interval of individual predicted values of M conditional on each A would lead to the false positive identification of cartilage-specific probe sets at medium and high intensities.

Based on the M-A plots, one of the reviewers has suggested the following iterated logarithm approach for normalization. Let log_2_(log_2_R)-log_2_(log_2_G) be M and (log_2_(log_2_R)+log_2_(log_2_G))/2 be A to perform Lowess normalization. After normalization, for each comparison, linear quantile regression of M on A was fitted to the data. 39 probe sets were above the estimated 95% conditional percentile of M in all 10 tissue comparisons. In contrast, 37 probe sets were above the estimated 95% conditional percentile of M in all 10 tissue comparisons using the originally proposed log transformation method. There were 32 probe sets common in both approaches. However, the iterated logarithm approach failed to identify 3 well-established cartilage biomarkers, which could be identified by the single log transformation approach. One possible reason is that the iterated logarithm may not remove intensity-dependent bias as well as the single logarithm.

## Conclusion

Quantile regression is appropriate for the analysis of two color array experiments, especially for studies with only one replicate and hence highly limited quantifiable sources of experimental error. We used a nonparametric approach to reveal the relationship between A and quantiles of M and then applied the appropriate quantile regression (in this study, it is linear quantile regression with intercept and a linear term) to select genes with a high level of expression in specific tissue or tissue biomarkers.

## Methods

### Microarray experiments

Articular cartilage and eight comparative tissues (kidney, lung, lymph node, cerebellum, spleen, bladder, liver, and muscle) were collected from a two-year old donor horse. Placental villous and testis samples were obtained independently from other donor horses. Total RNA was isolated from all of these eleven tissues by a traditional guanidinium isothiocyanate and phenol/chloroform separation protocol for total RNA isolation. Dye-coupled probes from the articular cartilage and each of the 10 tissues individually (cartilage/kidney, cartilage/lung, cartilage/lymph node, cartilage/placental villus, cartilage/cerebellum, cartilage/spleen, cartilage/bladder, cartilage/testis, cartilage/liver, and cartilage/muscle) were then hybridized to a 9852 element equine-specific cDNA microarray. All hybridizations were performed in duplicate with a dye swap to eliminate possible dye bias. After the post-hybridization washes, each slide was then immediately scanned using a GenePix 4100A scanner and spot intensities were computed using GENEPIX 6.0 image analysis software (Axon Instruments/Molecular Devices). Following background correction, the median intensities of each pair of spots were Lowess normalized for each individual slide. The bad-flagged spots on each slide were removed from the analyses.

### Algorithm and analysis

The statistical model in this study is that the *τ *th conditional quantile of *Y*_*i *_is *X*_*i*_*β*(*τ*) where *Y*_*i *_is the observed M, *X*_*i *_= (1,*x*_*i*_) and *x*_*i *_are the A, *β*(*τ*) = (*β*_0_(*τ*), *β*_1_(*τ*))^*t*^. We have employed *τ *values 1, 5, 10, 20, 50, 80, 90, 95 and 99%. Another way of writing this model is *Y*_*i *_= *X*_*i*_*β*(*τ*) + *ε*_*i*_(*τ*) with *ε*_*i*_(*τ*) having *τ *th quantile zero. The parameter *β*(*τ*) can be estimated by solving the minimizing problem:

$$\begin{array}{cc} \hat{\beta }(\tau )=\arg\min_{\beta \in R^{2}}\sum_{i=1}^{n}\rho_{\tau }(Y_{i}-X_{i}\beta ), & \;0<\tau <1 \end{array}$$

where *ρ*_*τ*_(*z*) = *z*(*τ *- *I*(*z *< 0)) and *I*(.) is the indicator function. Based on the estimated $\hat{\beta }(\tau )$, the predicted *τ *th quantile of *Y *given covariate value *x*_*i *_is *X*_*i*_$\hat{\beta }(\tau )$.

## Authors' contributions

LH carried out the statistical analyses. MZ, AJS and ACB supervised the study. WZ and JNM carried out the molecular genetics studies. All authors contributed to the writing of this manuscript. All authors read and approved the final manuscript.
